# The Future of Fascia—A Scoping Review on Emerging Research Trends and Recommended Sample Sizes and Effect Sizes in Fascia Studies

**DOI:** 10.3390/ijms26188871

**Published:** 2025-09-11

**Authors:** Grzegorz Zieliński

**Affiliations:** Department of Sports Medicine, Medical University of Lublin, 20-093 Lublin, Poland; grzegorz.zielinski@umlub.pl

**Keywords:** future, fascia, sample sizes, effect sizes

## Abstract

The aim of this study was to identify future research trends in fascia-related investigations, as well as to develop new effect size thresholds for between-group differences, along with sample size calculations and statistical power estimations specific to fascial research. For the purposes of this study, the following databases were searched: PubMed, Scopus, and Web of Science. Two studies were included in the scoping review, and 31 meta-analyses were identified and used to calculate effect sizes and sample sizes. Future research on fascia will likely focus on its role in movement organisation and chronic pain, particularly in older adults. The advancement of modern imaging techniques and the integration of therapeutic approaches, such as manual therapy and movement-based interventions, may significantly impact the diagnosis and treatment of fascial dysfunctions. In future studies on fascia, effect sizes (Cohen’s *d*, and Hedges’ *g*) of 0.05, 0.15, and 0.40 should be adopted as thresholds for small, medium, and large effects, respectively. The minimum sample size was estimated at *n* = 60, as the suggested number to detect large effect sizes with 60% statistical power. The required sample size is expected to increase depending on other study parameters.

## 1. Introduction

Fascia is one of the most underappreciated yet fundamentally important tissues of the musculoskeletal system [1,2]. Its anatomical definition has evolved over the past 400 years [3]. For a long time, it was regarded merely as a passive structure supporting muscles and organs. However, contemporary research has shown that it plays a significant role in force transmission, proprioception, regulation of muscle tension, inflammatory processes, and pain generation [1,4,5].

Fascia forms a continuous, three-dimensional network of connective tissue that permeates and envelops all components of the body—from muscles and blood vessels to internal organs [5,6]. Its dynamic cellular and molecular organisation makes it both a mechanical and functional organ, responsive to biomechanical and biochemical stimuli [4,5,7].

At the cellular level, fascia is composed predominantly of fibroblasts, myofibroblasts, and macrophages, which are responsible for the production and remodelling of the extracellular matrix (ECM) [8,9,10]. Fibroblasts regulate ECM homeostasis by synthesising collagen, elastin, fibronectin, and glycosaminoglycans, ensuring tissue elasticity and resilience [11,12]. Myofibroblasts, through alpha-smooth muscle actin (α-SMA) expression, generate contractile forces that may contribute to chronic changes in fascial tension and pain [13,14]. At the molecular level, the structure and function of fascia are primarily determined by the composition and orientation of collagen fibres (mainly types I and III), as well as by the hydration status of the ECM. The degree of hydration significantly affects the gliding capacity of the tissue and its ability to adapt to mechanical forces [15,16,17].

Despite the growing interest in fascial research, significant knowledge gaps remain. The existing literature is fragmented and often statistically heterogeneous. Most effect size analyses are based on frameworks developed by Jacob Cohen (1923–1998), an American psychologist and statistician [18]. There is also a lack of dedicated metrics capable of capturing the variability and plasticity of fascial tissue in response to various interventions—manual, movement-based, or pharmacological. Current measures are often insufficient to reflect the complex biomechanical and biochemical nature of fascia. Existing measures often fail to reflect fascia’s biomechanical and biochemical complexity. Recent studies also show that effect size benchmarks vary considerably across research domains [19,20,21,22,23,24]. This highlights the need for new, domain-specific thresholds tailored to fascial research.

Accordingly, developing effect size measures that incorporate both biomechanical and molecular variables is essential. Advanced study designs should also include sample size calculations based on expected effect sizes and clinically meaningful changes.

Only through such methodological refinement will it be possible to conduct high-quality studies with strong cognitive and clinical relevance, enabling a deeper understanding of fascial function and its role in the pathomechanisms of pain and movement dysfunctions, from the molecular level to the tissue level.

The aim of this study was to identify future research trends in fascial investigations and to propose new effect size thresholds, sample size guidelines, and statistical power estimations specific to this field.

## 2. Materials and Methods

### 2.1. Scoping Review

The scoping review was conducted in accordance with the Preferred Reporting Items for Systematic reviews and Meta-Analyses extension for Scoping Reviews (PRISMA-ScR) guidelines [25]. The review protocol was registered under DOI: https://doi.org/10.17605/OSF.IO/AJVTQ.

Three databases (PubMed, Scopus, Web of Science) were searched for publications between 1 January 2020 and 1 July 2025.

The search strategy was based on the presence of the keywords “fascia” and “future” in the title of publications. The initial search across all databases yielded 2075 results. After applying a publication date filter to include only studies from the past five years, the number of results was reduced to 734 articles. Following full-text screening and duplicate removal, two articles met the inclusion criteria.

Given the limited number of eligible studies, an additional Web of Science search using the Highly Cited Papers filter (as of 29 July 2025) identified 17 records, of which two further studies were included.

### 2.2. Project on Estimating Effect Sizes and Required Sample Sizes in the Context of Group Differences in Fascia Research

The project aimed at determining effect sizes and estimating the required sample sizes for investigating group differences in fascia-related studies was carried out in several stages.

Initially, a detailed analysis plan was registered, including both the literature search strategy and the approach to data analysis (https://doi.org/10.17605/OSF.IO/2QMSH). A systematic search of the Web of Science (23 July 2025) identified 330 eligible records. The search used the terms “fascia” (All Fields) and “meta” (All Fields), without restricting journal selection.

After full-text screening, 47 studies remained. Exclusions were applied as follows:

- Nine studies used effect size metrics other than Cohen’s d/SMD or MD;
- One study was retracted;
- Six studies lacked sufficient data.

Ultimately, 31 studies were included (listed in the Supplementary Materials Table S1). For studies reporting unstandardised mean differences, the standardised mean difference (SMD) was calculated using pooled standard deviations or, when necessary, estimated from reported means, errors, and sample sizes. Reported SMDs were retained, and small-sample bias was corrected by converting Cohen’s d to Hedges’ *g*. Where Hedges’ *g* was already provided, it was used directly [19,22].

Statistical analyses were performed in R using a custom script applied in earlier research [19,20,21,22,23,26]. Diagnostic tables assessed potential effect size inflation by plotting effect size estimates against their standard errors, allowing identification of deviations from population values. This approach helps detect bias due to publication practices, selective reporting, or low statistical power.

To aid interpretation, significance zones were colour-coded: orange (0.10 > *p* > 0.05) and red (0.05 > *p* > 0.01). Clustering in these areas may indicate inflation bias, suggesting reported effects exceed true population values [19,20,22,23]. Such analyses are especially relevant in meta-research, enabling critical evaluation of the reliability of aggregated evidence.

To estimate required sample sizes, a priori power analyses were performed using a two-tailed α = 0.05. A two-group model consistent with the independent samples *t*-test was adopted. Sample sizes were estimated for power levels of 60%, 70%, 80%, and 90%, providing benchmarks for moderate, good, and high sensitivity [19,20,22,23]. These thresholds align with methodologies in psychology and biomedical sciences [21,24]. The use of multiple power levels offers flexibility, particularly where recruiting large samples is challenging.

All statistical analyses were conducted using the R programming language (version 4.3.3; R Core Team, 2024) within a Windows 11 Pro 64-bit environment (build 22631). Statistical analyses were performed using dedicated R packages, including pwr [27], ggplot2 [28], report [29], metafor [30], numDeriv [31], cowplot [32], readxl [33], dplyr [33], matrix [34] and psych [35].

## 3. Result

### 3.1. Future Directions: Findings from the Scoping Review

Stecco et al. [36] introduced the concept of the myofascial unit as a fundamental element of motor control. This model emphasises the interaction between muscles, fascia, and intramuscular connective tissue in generating movement. Dysfunction in one component can impair the others, reducing functional capacity. By moving beyond the traditional view of muscles as the sole organisers of motion, this framework provides a more comprehensive explanation of myofascial pain and proprioceptive dysfunction and offers new diagnostic and therapeutic opportunities.

Du et al. [37] examined the role of fascia in chronic pain, particularly among older adults. Their bibliometric analysis of 744 publications (2013–2022) identified major research centres, leading authors, and thematic directions. Fascia is increasingly recognised as central to the development and persistence of pain, with modern imaging techniques facilitating detection of pathological changes. Key topics include myofascial pain syndrome, nerve blocks, and interventional therapies such as yoga, acupuncture, and tai chi.

Together, these studies highlight a growing research interest in fascia with significant clinical implications. Stecco et al. [36] provide an innovative framework for understanding movement organisation, while Du et al. [37] underscore fascia’s role in chronic pain within an ageing population. Future research is expected to integrate manual therapies, movement-based interventions, and neurophysiological methods, potentially transforming pain management strategies.

### 3.2. Estimating Effect Sizes and Required Sample Sizes in the Context of Group Differences in Fascia Research

**Figure 1:**
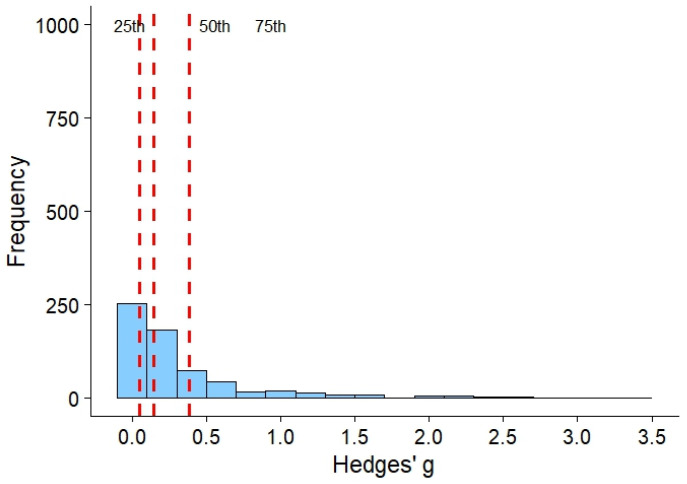
Distribution of Hedges’ *g* effect sizes with dashed red lines representing the 25th, 50th, and 75th percentiles, corresponding to small (*g* = 0.05), medium (*g* = 0.15), and large (*g* = 0.40) effect sizes.

The first (25%), second (50%), and third (75%) percentiles for group differences correspond to 0.05, 0.15, and 0.40, respectively (Table 1 and Figure 1).

**Figure 2:**
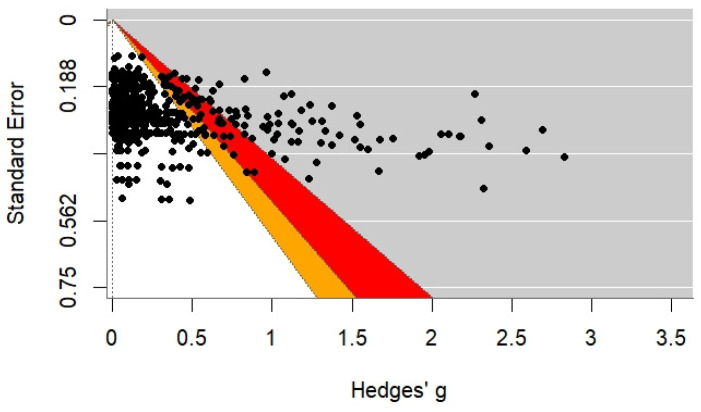
Funnel plot results.

Additionally, the funnel plot analysis did not reveal an overrepresentation of marginally significant results (*p*-values in the range of 0.05–0.01, marked in red) or near-significant results (*p*-values between 0.10 and 0.05, marked in orange). This distribution suggests a low risk of inflated findings, including publication bias or statistical manipulation (so-called *p*-hacking). The overall distribution appears fair and representative of the true population effect sizes. In terms of proportions, 14.34% of the results were in grey, 6.83% in red, 3% in orange, and 75.83% in white (Figure 2).

One of the key aims of this study was to estimate the number of participants required to achieve a given level of statistical power for a presumed effect size. All analyses were conducted using a two-tailed significance level of α = 0.05, in accordance with commonly accepted standards in scientific research. For each scenario, the required sample size was calculated to achieve statistical power levels of 60%, 70%, 80%, and 90%—corresponding to moderate, good, and high sensitivity to detect a true effect, if one exists. Further details are provided in Table 2.

## 4. Discussion

The aim of this study was to identify future research trends in fascial investigations and to propose new effect size thresholds, sample size guidelines, and statistical power estimations specific to this field.

Future research on fascia will primarily focus on its role in the organisation of movement and in chronic pain, particularly among the elderly. Understanding how fascia influences the functioning of the musculoskeletal system may open new diagnostic and therapeutic avenues. This is especially important in the context of an ageing population, which will lead to increasing demand for professionals specialising in the treatment of the locomotor apparatus [1,38,39].

Furthermore, future studies are likely to place greater emphasis on the integration of diverse therapeutic approaches. Manual therapies, such as fascial manipulation, and movement-based interventions—e.g., stretching exercises or functional training—will be analysed in terms of their effectiveness in improving fascial flexibility and function [40,41,42]. Understanding the mechanisms underlying these therapies will enable the optimisation of treatment and its adaptation to the individual needs of patients. In this context, appropriate statistical guidelines become even more essential.

Recent evidence suggests that effect sizes in group studies are smaller than Cohen’s conventional thresholds (0.2, 0.5, 0.8) [43]. Percentile-based analyses indicate approximate benchmarks of 0.05, 0.15, and 0.40 for small, medium, and large effects. Most observed effects fall below Cohen’s smallest threshold, suggesting earlier studies may have overestimated effect magnitude and clinical relevance. This calls for larger samples, more precise measurements, and more realistic power planning.

Recent data suggest that actual effect sizes in group studies are significantly smaller than the conventional thresholds proposed by Cohen (small = 0.2; medium = 0.5; large = 0.8) [43]. Percentile-based analyses show that intergroup differences correspond approximately to 0.05 (25th percentile), 0.15 (50th percentile), and 0.40 (75th percentile). This means that most observed effects in research lie below even the smallest of Cohen’s thresholds. This has serious implications for future research and the scientific discipline as a whole. Above all, it compels researchers to reconsider how the significance of effect sizes is interpreted. Many earlier studies may have overestimated the magnitude of effects, leading to inaccurate conclusions or inflated assessments of the practical significance of findings.

For future research, this necessitates larger sample sizes, more precise measurement methods, and more realistic planning of statistical power. This shift also affects scientific publishing—results showing smaller, yet reliable effects must be treated with greater respect, even if they no longer meet traditional “thresholds”. The entire field will need to revise its standards of result interpretation to more accurately reflect the nature of the phenomena under study.

As highlighted in Table 2, large study groups are required particularly when attempting to detect small effects, which presents substantial challenges in meeting such demands. Conversely, when aiming to detect large effects, the necessary group sizes are much more feasible and represent more standard sample sizes [19,20,22,23].

From a clinical perspective, the proposed thresholds and sample size guidelines could support more robust trial design in fascial research. For example, adopting lower effect size benchmarks (e.g., 0.05 or 0.15) may help clinicians and researchers detect subtle, yet clinically meaningful improvements in pain, mobility, or function that traditional thresholds would overlook. In practical terms, this implies that rehabilitation or manual therapy studies targeting fascia may need to recruit larger patient groups than is customary, especially when studying chronic pain in older adults. Moreover, preliminary thresholds can guide feasibility studies by allowing researchers to balance clinical relevance with realistic recruitment capacities. Importantly, these guidelines should not be applied rigidly; rather, they should serve as orientation points that can be adapted to specific clinical populations, therapeutic modalities, and study objectives.

Finally, it is worth emphasising that effect sizes should serve as a tool to support clinical analysis and reasoning. However, they should not replace the researcher’s judgement or critical evaluation of the results [19,44].

This study has several limitations. The primary limitation is the relatively small sample included in the scoping review, although it nonetheless enabled the estimation of future research trends. A typical limitation concerning the estimation of effect sizes is the risk of bias [20,23]. It is assumed that the authors of the original meta-analyses accurately reported the effect sizes obtained. Should an error have occurred at this stage, it could have affected the accuracy of the newly proposed effect size thresholds. Nevertheless, the funnel plot analysis indicated that this type of bias was minimal.

## 5. Conclusions

Future research on fascia will likely focus on its role in movement organisation and chronic pain, particularly in older adults. The advancement of modern imaging techniques and the integration of therapeutic approaches, such as manual therapy and movement-based interventions, may provide preliminary insights into the diagnosis and treatment of fascial dysfunctions.

In future studies on fascia, it has been tentatively suggested that effect sizes (Cohen’s *d*, and Hedges’ *g*) of 0.05, 0.15, and 0.40 could serve as preliminary reference points for small, medium, and large effects, respectively. The minimum sample size was estimated at *n* = 60, as the suggested number to detect large effect sizes with 60% statistical power. However, these thresholds should be regarded as provisional, and the required sample size is likely to increase depending on additional study parameters and methodological considerations.

## Figures and Tables

**Table 1:** Percentiles associated group differences (Hedges’ *g*).

Percentile	Hedges’ *g*
5	0.00
10	0.01
15	0.02
20	0.03
25	0.05
30	0.07
35	0.09
40	0.10
45	0.13
50	0.15
55	0.18
60	0.21
65	0.29
70	0.34
75	0.40
80	0.53
85	0.69
90	1.02
95	1.67

**Table 2:** Sample sizes required to achieve various levels of statistical power in the group differences.

Effect Size	Statistical Power			
	60%	70%	80%	90%
Small (*g* = 0.05)	4073	5132	6525	8735
Medium (*g* = 0.15)	417	526	668	984
Large (*g* = 0.40)	60	77	98	131

## Data Availability

Not applicable.

## Supplementary Materials

The following supporting information can be downloaded at: https://www.mdpi.com/article/10.3390/ijms26188871/s1.
